# Functional Status of Pancreatic *α* and *β* Cells in Type 2 Diabetes Mellitus Patients with Different Plasma Triglyceride Levels: A Retrospective Analysis

**DOI:** 10.1155/2021/9976067

**Published:** 2021-08-17

**Authors:** Hang Guo, Chunlei Ma, Xiaoming Wu, Congqing Pan

**Affiliations:** ^1^NHC Key Laboratory of Hormones and Development, Tianjin Key Laboratory of Metabolic Diseases, Chu Hsien-I Memorial Hospital & Tianjin Institute of Endocrinology, Tianjin Medical University, Tianjin, China; ^2^Department of Urology, Tianjin 4th Center Hospital, The Fourth Central Hospital Affiliated to Nankai University, Tianjin 300140, China

## Abstract

**Objective:**

To investigate the functional status of pancreatic *α* and *β* cells in Type 2 diabetes mellitus (T2DM) patients with different plasma triglyceride (TG) levels. TG levels can be prognostic markers for T2DM.

**Methods:**

A total of 328 patients with T2DM were divided into three groups according to different TG levels: the TGL group: TG < 1.7 mmol/L; TGM group: 1.7 mmol/L ≤ TG < 2.3 mmol/L; and TGH group: TG ≥ 2.3 mmol/L. An oral glucose tolerance test (OGTT), insulin release test, and glucagon release test were performed in each patient. The changes of glucagon, glucagon/insulin ratio, early insulin secretion index (Δ*I*_30_/Δ*G*_30_), and area under the insulin curve (AUC_I_) were compared among each group. Also, the correlations between glucagon and pancreatic *β*-cell function, glycosylated hemoglobin (HbA1c), and other indices were analyzed.

**Results:**

With the increase of TG, the fasting and postprandial glucagon levels, the glucagon/insulin ratio, and the area under the glucagon curve (AUC_G_) presented an increasing trend. The homeostasis model assessment of insulin resistance (HOMA–IR) of the TGH group was significantly increased compared to the TGL and TGM groups. In addition to the increase in TG levels, the insulin sensitivity index (ISI), homeostasis model assessment for *β*-cell function index (HOMA-*β*), Δ*I*_30_/Δ*G*_30_, and AUC_I_ displayed a reducing trend. Glucagon was negatively correlated with Δ*I*_30_/Δ*G*_30_, high-density lipoprotein (HDL), HOMA-*β*, body mass index (BMI), ISI, and AUC_I_ (*P* < 0.05) and positively correlated with fasting blood glucose (FPG), AUC_G_, HOMA-IR, HbA1c, duration, TG, low-density lipoprotein (LDL), and total cholesterol (TC) (*P* < 0.05).

**Conclusion:**

Hypertriglyceridemia aggravated the dysfunction of pancreatic *α* and *β* cells. A reasonable control of the TG level makes it easier for blood glucose to reach the standard.

## 1. Background

Diabetes has become a global chronic noninfectious disease and a public health problem of worldwide concern [1]. The prevalence of T2DM is increasing year by year. The number of people with diabetes worldwide was estimated at almost 400 million in 2013 and is projected to increase approximately up to 600 million by 2035 [2].

In 2017, the prevalence of diabetes in China was 11.2%, making it the country with the largest number of diabetes patients [3]. As we all know, the complications of diabetes will involve multiple organs of the body and increase the risk of death. Diabetes not only reduces the quality of life of patients but also causes huge economic burden. As a result, the country's medical resources are consumed greatly, making diabetes a huge burden for people all over the world [4].

Insulin resistance (IR) and impaired secretion of pancreatic *β* cells are the main pathophysiological mechanisms of T2DM [5]. Unger et al. put forward the “bihormonal theory,” which believed that not only abnormal function of pancreatic islet *β* cells but also abnormal function of *α* cells existed in diabetic patients and insulin resistance/lack and absolute/relative increase of glucagon jointly lead to diabetes [6]. Glycemic balance is regulated by the glucagon secreted by pancreatic *α* cells and insulin secreted by pancreatic *β* cells [7]. In recent years, the effects of glucagon on T2DM have attracted much attention from medical researchers [8].

Previous studies have found that T2DM is associated with abnormalities in lipid metabolism such as high fasting and postprandial blood levels of TG, high concentrations of nonesterified fatty acids, and low HDL concentrations [9]. In addition, a study has reported that hyperlipidemia can increase glucagon in patients with T2DM [10]. However, the relationship between blood lipid levels and the function of pancreatic *α* and *β* cells has been rarely studied. This study retrospectively analyzed the functional changes occurring in pancreatic *α* and *β* cells in T2DM patients with different plasma TG levels.

## 2. Participants and Methods

### 2.1. Participants

A total of 328 patients with T2DM admitted to Tianjin Medical University Chu Hsien-I Memorial Hospital between August 2018 and August 2020 were enrolled for the study. The study was approved by the Ethics Committee of Chu Hsien-I Memorial Hospital of Tianjin Medical University (No. DXBYYhMEC2020-35). This patient group included 168 males and 160 females with an average age of 54.9 ± 10.4 years. T2DM was diagnosed according to the diagnostic criteria laid by the World Health Organization (1999): (FBG ≥ 7.0 mmol/L and 2-hour postprandial blood glucose (2hPBG) ≥ 11.1 mmol/L). The enrolled patients were devoid of using lipid-regulating drugs or insulin preparations from six months prior to hospitalization. The patients were also inhibited from insulin secretagogues three days before the OGTT test.

#### 2.1.1. Exclusion Criteria

Patients suffering from severe liver and kidney dysfunction, tumor, anemia, acute cardiovascular and cerebrovascular events, acute and chronic inflammation, stress state, ketosis or hypertonic coma, gestational diabetes, secondary diabetes, and other endocrine, metabolic diseases were excluded from the study.

#### 2.1.2. Grouping

According to the Guidelines for the Prevention and Treatment of Dyslipidemia in Chinese Adults (2010), the subjects were divided into three groups according to different TG levels: the TGL group: TG < 1.7 mmol/L; TGM group: 1.7 mmol/L ≤ TG < 2.3 mmol/L; and TGH group: TG ≥ 2.3 mmol/L.

### 2.2. Methods

#### 2.2.1. Sampling Strategy

The venous blood was collected from the peripheral forearm of the enrolled patients after an overnight fast of 8–12 hours. TC, TG, HDL, LDL, liver, kidney function tests, and other biochemical indices were measured using the Hitachi 7180 automatic biochemical analyzer. HBA1c was determined by high-pressure liquid chromatography. An OGTT, insulin release test, and glucagon release test were performed. Patients were inhibited from using insulin secretagogue three day before the blood draw. 75 g glucose was administered orally after an overnight fast of 12 hours. Venous blood from the forearm was collected at 0, 30, 60, 120, and 180 mins, respectively, to evaluate the FBG and 2hPBG, insulin and glucagon levels. The glucose oxidase method was used to assess glucose levels. Insulin was measured by electrochemical luminescence (Roche, Germany), and glucagon was measured by nonequilibrium radioimmunity.

#### 2.2.2. Indicators and Measurements

The area under the curve of insulin and area under the curve of glucagon were calculated using the following formula: AUC_I_ = 15 × fasting insulin level + 30 × (insulin level at 30 mins + insulin level at 180 mins) + 45 × insulin level at 60 mins + 60 × insulin level at 120 mins; AUC_G_ = 15 × fasting glucagon level + 30 × (glucagon level at 30 mins + glucagon level at 180 mins) + 45 × glucagon level at 60 mins + 60 × glucagon level at 120 mins. Homeostasis model assessment of insulin resistance (HOMA-IR = FPG × fasting insulin (FI)/22.5) was used to evaluate IR, insulin sensitivity index (ISI = ln (FI × (fasting glucagon) FG)) was used to evaluate insulin sensitivity, and homeostasis model assessment for *β*-cell function index (HOMA-*β* = 20 × FI/(FPG-3.5)) was used to reflect the basal insulin secretion. Early-phase insulin secretion was evaluated by the ratio of the net increase in insulin and glucose after 30 mins of glucose load (Δ*I*_30_/Δ*G*_30_), and the AUC_I_ was used to evaluate the second-phase insulin secretion.

### 2.3. Statistical Processing

SPSS 18.0 software was used for statistical analysis. Normal distribution data were expressed as *x̅* ± *s*, and nonnormal distributions were normalized by natural logarithmic transformation before statistical analysis. The comparison between different groups and different time points used repeated-measurement data analysis of variance. The comparison among multiple groups was performed by one-way analysis of variance (ANOVA) and the least significant difference (LSD) test. Pearson correlation analysis was used to analyze the correlation between glucagon and other parameters. *P* < 0.05 was considered statistically significant.

## 3. Results

### 3.1. Comparison of Patients' General Information

There were no significant differences in age, gender composition, course of the disease, BMI, systolic blood pressure, diastolic blood pressure, and hemoglobin among the three groups. HbA1c, LDL, and FPG levels of the TGH group were significantly higher than those of the TGL group and TGM group. The high-density lipoprotein cholesterol was significantly increased in the TGM and TGH groups (*P* < 0.05) compared to the TGL group. A significant difference (*P* < 0.05) was observed for TC among the three groups (Table 1).

### 3.2. Glucagon Levels (in Fasting State and after Glucose Load) and Glucagon/Insulin Ratio in Patients with Different TG Levels

**Figure 1:**
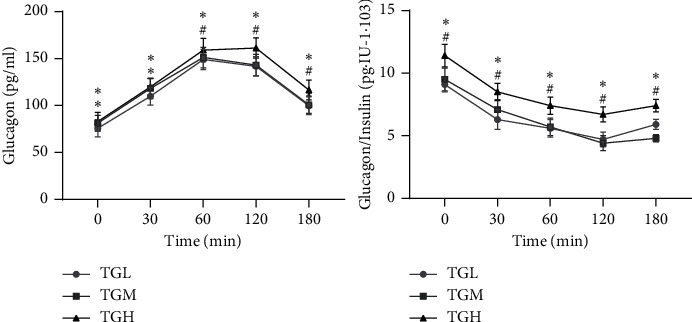
Glucagon and glucagon/insulin before and after OGTT among patients with different HbA1c. TGL: TG < 1.7 mmol/L; TGM: TGM 1.7 ≤ TG < 2.3 mmol/L; and TGH: TG ≥ 2.3 mmol/L. ^*∗*^*P* < 0.05 versus TCL; ^#^*P* < 0.05 versus TCM.

With the increase in TG levels, the three groups' glucagon levels increased at all time points. The glucagon level of the TGH group was significantly higher than that of the TGL group at all other time points (fasting (82.9 ± 9.9 pg/ml vs. 75.8 ± 9.2 pg/ml); 30 mins (119.9 ± 8.6 pg/ml vs. 109.0 ± 9.3 pg/ml); 60 mins (159.1 ± 12.4 pg/ml vs. 149.0 ± 10.8 pg/ml); 120 mins (161.3 ± 10.7 pg/ml vs. 141.8 ± 10.2 pg/ml); and 180 mins (116.6 ± 10.5 pg/ml vs. 99.8 ± 9.4 pg/ml), *P* < 0.05). Except for fasting glucagon and glucagon at 30 mins higher than the TGL group (*P* < 0.05), the TGM group had no statistically significant difference compared with the TGL group at other time points. Except for glucagon at 30 mins and glucagon at 120 mins higher than the TGM group (*P* < 0.05), the TGH group had no statistically significant difference compared with the TGM group at other time points (Figure 1(a)). With the increase of TG level, the glucagon/insulin ratio showed an overall increasing trend in fasting state and at each time point after the glucose load. The TGH group was significantly higher than the TGM and TGL groups simultaneously, and there was no statistical difference between the TGL and TGM groups (Figure 1(b)).

### 3.3. Comparison of the AUC_G_ and the Function of Pancreatic *β* Cells in T2DM Patients with Different TG Levels

**Figure 2:**
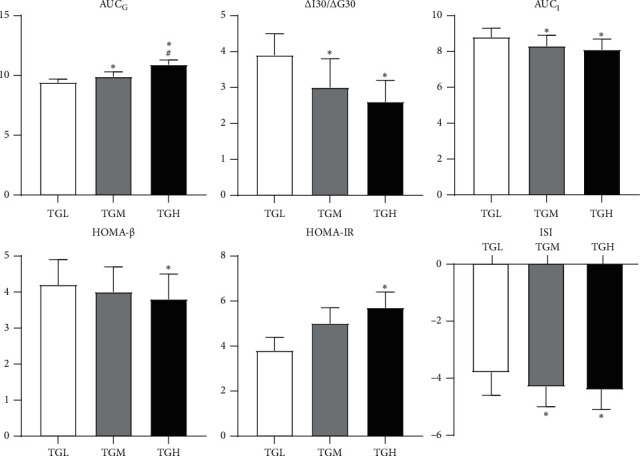
Islet *β*-cell function and AUCG among patients with different HbA1c levels. TGL: TG < 1.7 mmol/L; TGM: TGM 1.7 ≤ TG < 2.3 mmol/L; and TGH: TG ≥ 2.3 mmol/L. AUC_G_: area under the curve of glucose; Δ*I*_30_/Δ*G*_30_: ratio of insulin increment and glucose increment 30 min after glucose load; AUC_I_: area under the curve of insulin; HOMA-*β*: homeostasis model assessment of *β*-cell function; HOMA-IR: homeostasis model assessment of insulin resistance index; and ISI: insulin sensitivity index. ^*∗*^*P* < 0.05 versus TCL; ^#^*P* < 0.05 versus TCM.

The AUC_G_ exhibited an increasing trend with the increase of TG, characterized by statistical differences among all the three groups (*P* < 0.05). Besides, an increase in TG resulted in a significant increase of HOMA-IR in the TGH group when compared to TGM and TGL groups, and contrary results were achieved for the parameters of ISI, HOMA-*β*, Δ*I*_30_/Δ*G*_30_, and AUC_I_ (*P* < 0.05) (Figure 2).

### 3.4. Analysis of the Correlation Coefficient between Glucagon and Related Indices

Glucagon was negatively correlated with Δ*I*_30_/Δ*G*_30_, HDL, HOMA-*β*, BMI, ISI, and AUC_I_ with *r* values −0.229, −0.165, −0.153, −0.151, −0.146, and −0.136, respectively (*P* < 0.05). Glucagon was positively correlated with FPG, AUC_G_, HOMA-IR, HbA1c, duration, TG, LDL, and TC, with *r* values 0.545, 0.476, 0.325, 0.273, 0.193, 0.141, 0.111, and 0.066 (*P* < 0.05) (Table 2).

## 4. Discussion

The elevation of blood glucose levels in T2DM patients is caused by the secretory dysfunction of pancreatic *α* and *β* cells [11]. Consequently, the impaired insulin secretion and excessive glucagon secretion leads to an unusual rise in glucagon/insulin ratio [12]. This condition aggravates hyperglycemia. Research by Cook et al. reported that greater than 50% of T2DM patients in the United States suffered comorbidity with varying degrees of dyslipidemia [13]. Furthermore, the CCMR-3B study performed in China involving more than 20,000 T2DM patients from 107 hospitals across the country revealed that about 42% of them were associated with dyslipidemia [14].

The prevention and treatment of dyslipidemia in diabetic patients have become a trending topic of recent research with evidence of a high prevalence of dyslipidemia [15]. The characteristic pathological features of T2DM include insulin resistance and impaired insulin secretion [16]. It has been found that the disorder in lipid metabolism can aggravate IR in T2DM patients and lipid toxicity can aggravate the functional decline of pancreatic *β* cells [17]. The current study results showed that the increase of plasma TG level caused an increasing trend in the insulin resistance index of pancreatic *β* cells, while the ISI showed a decreasing trend in T2DM patients. Simultaneously, the HOMA-*β* and early-phase insulin secretion index were significantly decreased, which was consistent with the results of previous studies. In hypertriglyceridemia, the TG and free fatty acids are deposited in nonadipose tissue, resulting in reduced insulin and IR biological effects. Under pathological conditions, TG and free fatty acids can be deposited in pancreatic islet tissues and directly damage pancreatic *β* cells, leading to insulin secretion disorder [18].

This study focused on the effect of TG level on the function of pancreatic *α* and *β* cells. In recent years, it has been found that glucagon secreted by pancreatic *α* cells also plays a vital role in the development and T2DM progression [19]. In this study, T2DM patients were grouped according to different TG levels. The results indicated that the increase in plasma TG levels increased the patients' glucagon levels and AUC_G_. This indicated that the higher TG levels induced the secretion of glucagon in pancreatic *α* cells. It was considered that this might be related to the dysfunction of pancreatic *α* cells in these patients. In hypertriglyceridemia, free fatty acids lead to high glucagon secretion and increase TG accumulation and free fatty acids in pancreatic *α* cells. It has been speculated that continuous exposure to free fatty acids may reduce AMP-activated kinase protein activity and increase the accumulation of TG, leading to changes in the insulin signal of pancreatic *α* cells and excessive secretion of glucagon [20]. In addition, it has been reported that pancreatic *α*-cell dysfunction may be mediated by the accumulation of TG-rich lipoproteins [21, 22]. Due to poor activation of GABA-A receptors of pancreatic *α* cells, these lipoproteins can interfere with glucagon secretion [23].

In addition to the direct effects mentioned above, the effect of blood lipids on glucagon secretion may have indirect effects. In T2DM patients, the high glucose inhibition effect can directly lead to a high secretion state of glucagon, further aggravating blood glucose disorder, forming a vicious cycle of mutual promotion of blood glucose, blood lipids, and glucagon.

The glucagon/insulin ratio is an important factor in determining blood glucose concentration. Due to impaired insulin secretion and excessive glucagon secretion in T2DM patients, the glucagon/insulin ratio is abnormally increased, thus exacerbating the hyperglycemia [24]. In this study, it was found that the glucagon/insulin ratio showed an overall increasing trend as the TG level increases in the fasting state and at each time point after the glucose load [25]. The TGH group showed consistent and significantly higher levels than TGM and TGL groups at the same time points. In hypertriglyceridemia, increased plasma free fatty acid levels lead to IR and pancreatic *β*-cell secretion deficiency, resulting in decreased insulin levels. Furthermore, it also affects the pancreatic *α*-cell function and leads to glucagon hypersecretion, resulting in increased glucagon/insulin ratio [20].

The medical records collected in this study belonged to Tianjin, China. The patient population may not represent the whole T2DM patient population.

## 5. Conclusions

From the present study, it can be concluded that when treating T2DM, monitoring the plasma TG levels is equally important as that of blood glucose levels. Reasonable control of the TG level is conducive to regulating the glucagon and insulin ratio to achieve a more effective standard blood glucose control.

## Figures and Tables

**Table 1:** Comparison of clinical data among patients with different TG levels.

	TGL (*n* = 114)	TGM (*n* = 101)	TGH (*n* = 113)	*p* value
Age (year)	55.1 ± 9.8	54.0 ± 9.8	53.9 ± 10.1	>0.05
Female (*n*)	45	36	41	>0.05
Duration (year)	8.6 ± 2.0	8.4 ± 2.4	7.9 ± 2.2	>0.05
BMI (kg/m^2^)	28.0 ± 2.8	27.1 ± 3.2	28.6 ± 2.2	>0.05
FPG (mmol/L)	7.4 ± 1.5	7.5 ± 1.9	7.9 ± 1.7^*∗*^	<0.05
SBP (mmHg)	131.7 ± 18.0	131.2 ± 16.4	131.4 ± 16.2	>0.05
DBP (mmHg)	77.9 ± 9.0	77.2 ± 12.0	79.4 ± 10.5	>0.05
TC (mmol/L)	4.9 ± 0.9	5.2 ± 1.1^*∗*^	5.5 ± 1.4^*∗*#^	<0.05
HDL (mmol/L)	1.4 ± 0.4	1.3 ± 0.3^*∗*^	1.3 ± 0.3^*∗*^	<0.05
LDL (mmol/L)	3.0 ± 0.7	3.3 ± 0.9	3.7 ± 1.0^*∗*^	<0.05
HbA1c (%)	8.3 ± 2.4	8.2 ± 1.8	8.6 ± 2.6^*∗*^	<0.05
HGB (g/L)	145.4 ± 16.1	144.0 ± 20.2	143.2 ± 16.6	>0.05

TGL: TG < 1.7 mmol/L; TGM: TGM 1.7 ≤ TG < 2.3 mmol/L; TGH: TG ≥ 2.3 mmol/L; BMI (kg/m^2^): body mass index; TC: total cholesterol; HDL: high-density lipoprotein; LDL: low-density lipoprotein; SBP: systolic blood pressure; DBP: diastolic blood pressure; ^*∗*^*P* < 0.05 versus TCL; ^#^*P* < 0.05 versus TCM.

**Table 2:** Correlation analysis between glucagon and related parameters.

	FBG	AUC_G_	HOMA-IR	HbA1c	Δ*I*_30_/Δ*G*_30_	Duration	HDL	HOMA-*β*	BMI	ISI	TG	AUC_I_	LDL	TC
*r*	0.545	0.476	0.325	0.273	−0.229	0.193	−0.165	−0.153	−0.151	−0.146	0.141	−0.136	0.111	0.066
*p*	<0.05	<0.05	<0.05	<0.05	<0.05	<0.05	<0.05	<0.05	<0.05	<0.05	<0.05	<0.05	<0.05	＞0.05

FPG: fasting plasma glucose; AUC_G_: area under the curve of glucose; HOMA-IR: homeostasis model assessment of insulin resistance index; HbA1c: glycosylated hemoglobin; Δ*I*_30_/Δ*G*_30_: ratio of insulin increment and glucose increment 30 min after glucose load; HOMA-*β*: homeostasis model assessment of *β*-cell function; BMI: body mass index; ISI: insulin sensitivity index; AUC_I_: area under the curve of insulin; duration: duration of diabetes. TGL: TG < 1.7 mmol/L; TGM: TGM 1.7 ≤ TG < 2.3 mmol/L; TGH: TG ≥ 2.3 mmol/L. AUC_G_: area under the curve of glucose; Δ*I*_30_/Δ*G*_30_: ratio of insulin increment and glucose increment 30 min after glucose load; AUC_I_: area under the curve of insulin; HOMA-*β*: homeostasis model assessment of *β*-cell function; HOMA-IR: homeostasis model assessment of insulin resistance index; ISI: insulin sensitivity index.

## Data Availability

All data are freely available for scientific purpose.

## Abbreviations

TG: Plasma triglyceride
OGTT: Oral glucose tolerance test
AUC_I_: Area under the insulin curve
HbA1c: Glycosylated hemoglobin
AUC_G_: Area under the glucagon curve
ISI: Insulin sensitivity index
LDL: Low-density lipoprotein
TC: Total cholesterol
IR: Insulin resistance
FBG: Fasting blood glucose
SBP: Systolic blood pressure
DBP: Diastolic blood pressure
BMI: Body mass index
HOMA-IR: Homeostasis model assessment of insulin resistance index
Δ*I*_30_/Δ*G*_30_: Ratio of insulin increment and glucose increment 30 min after glucose load
HOMA-*β*: Homeostasis model assessment of *β*-cell function.
